# Alteration of Epigenetic Modifiers in Pancreatic Cancer and Its Clinical Implication

**DOI:** 10.3390/jcm8060903

**Published:** 2019-06-24

**Authors:** Yu-Hsuan Hung, Ming-Chuan Hsu, Li-Tzong Chen, Wen-Chun Hung, Mei-Ren Pan

**Affiliations:** 1 National Institute of Cancer Research, National Health Research Institutes, Tainan 704, Taiwan; paganpoetry2005@gmail.com (Y.-H.H.); mchsu@nhri.edu.tw (M.-C.H.); leochen@nhri.edu.tw (L.-T.C.); hung1228@nhri.edu.tw (W.-C.H.); 2 Division of Hematology/Oncology, Department of Internal Medicine, National Cheng Kung University Hospital, Tainan 704, Taiwan; 3 Institute of Medicine, Kaohsiung Medical University, Kaohsiung 807, Taiwan; 4 Institute of Clinical Medicine, College of Medicine, Kaohsiung Medical University, Kaohsiung 807, Taiwan; 5 Center for Cancer Research, Kaohsiung Medical University, Kaohsiung 807, Taiwan; 6 Department of Medical Research, Kaohsiung Medical University Hospital, Kaohsiung 807, Taiwan

**Keywords:** pancreatic cancer, epigenetic regulation, SWItch/Sucrose Non-Fermentable (SWI/SNF) complex, histone methylation, synthetic lethality

## Abstract

The incidence of pancreatic cancer has considerably increased in the past decade. Pancreatic cancer has the worst prognosis among the cancers of the digestive tract because the pancreas is located in the posterior abdominal cavity, and most patients do not show clinical symptoms for early detection. Approximately 55% of all patients are diagnosed with pancreatic cancer only after the tumors metastasize. Therefore, identifying useful biomarkers for early diagnosis and screening high-risk groups are important to improve pancreatic cancer therapy. Recent emerging evidence has suggested that genetic and epigenetic alterations play a crucial role in the molecular aspects of pancreatic tumorigenesis. Here, we summarize recent progress in our understanding of the epigenetic alterations in pancreatic cancer and propose potential synthetic lethal strategies to target these genetic defects to treat this deadly disease.

## 1. Introduction

Pancreatic cancer is one of the leading causes of cancer-related death worldwide [1,2,3]. In addition to surgery [4,5], chemotherapy [3,5], and novel treatments [6] are required to improve therapeutic efficiency against pancreatic cancer. Biomarkers for pancreatic cancer have been widely studied [7,8] and have been clinically used for disease prediction [7] and therapeutic target identification [9], which has resulted in the development of next-generation sequencing and proteomic analysis techniques [8,9]. Genetic biomarkers in pancreatic cancer patients [9] may offer therapeutic benefits by revealing the downstream signaling pathways [10], but this benefit is also affected by the druggability of their protein products [11]. Conversely, biomarkers of epigenetic regulators may provide advantages for cancer treatment due to their versatile regulation of multiple genes [12]. Thus, researchers have focused on the biomarkers of epigenetic regulators with a high incidence rate in pancreatic cancer patients [8,9,13]. These biomarkers include the chromatin remodeler SWItch/Sucrose Non-Fermentable (SWI/SNF) members (AT-rich interaction domain 1A (*ARID1A*), SWI/SNF related, matrix associated, actin dependent regulator of chromatin, subfamily a, member 2 (*SMARCA2*), and a SWI/SNF related, matrix associated, actin dependent regulator of chromatin, subfamily a, member 4 (*SMARCA4*)), histone methylation regulators (lysine demethylase 6A (*KDM6A*), lysine methyltransferase 2C (*KMT2C*), and lysine methyltransferase 2D (*KMT2D*)), and epigenetic readers, including bromodomain and extraterminal domain (BET) proteins (bromodomain containing 2 (*BRD2*), bromodomain containing 3 (*BRD3*), bromodomain containing 4 (*BRD4*), and bromodomain testis associated (*BRDT*)). Table 1 shows the gene aliases listed in the NCBI database, and the importance of these genes in pancreatic cancer and in other gastrointestinal (GI) cancers are discussed in Table 2.

## 2. Expression and Mutation of SWI/SNF Genes in Pancreatic Cancer

**Figure 1:**
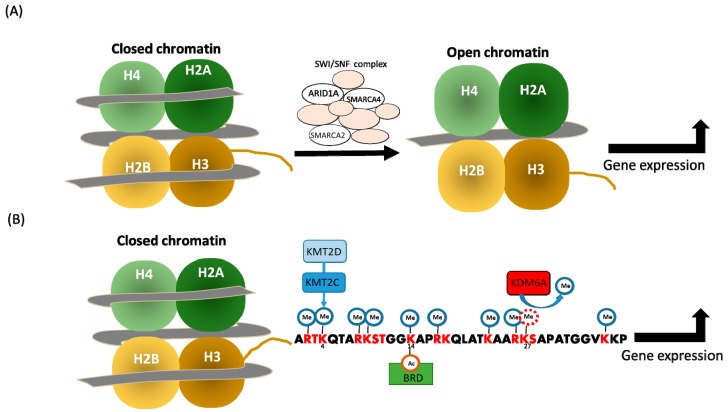
Epigenetic regulation of the SWI/SNF complex, lysine methyltransferase, and demethylase. (**A**) The SWI/SNF complex generates an open chromatin structure to initiate transcription. (**B**) The activation of H3K27m2/3 demethylase KDM6A, H3K4m1 methyltransferase KMT2D/KMT2C, and bromodomain and extraterminal domain (BET) proteins leads to transcriptional activation.

The epigenetic regulation of gene expression occurs through covalent modifications of DNA or histones, nucleosome positioning, and non-coding RNA, such as microRNA [60]. Therapeutics targeting the covalent modifications of DNA or histones have been approved for cancer treatment or are undergoing clinical trials in various neoplasia, including pancreatic cancer [61]. In addition, chromatin remodelers mobilize the nucleosome and regulate both chromatin accessibility/gene expression [62] and cancer progression [63]. Chromatin remodelers are categorized into the families of SWI/SNF, ISWI, Mi-2/NuRD, and INO80/SWR1, to identify histone lysine acetylation, nucleosome, histone lysine methylation, and actin-related factors, respectively [64] (Figure 1). The mutations prevailing in the SWI/SNF complex during pancreatic tumorigenesis [45,65] are discussed below:

### 2.1. ARID1A

ARID1A is a member of the SWI/SNF complex and is involved in transcriptional factor/cofactor/corepressor binding during chromatin remodeling [66]. While ARID1A is not required for pancreatic morphogenesis, its loss is associated with increased mucinous and pancreatic intra-epithelial neoplasia (PanIN) lesions [67]. ARID1A nonsense/missense mutations have been reported in tumors from patients with pancreatic cancer [40,41], and an in vitro or in vivo loss of ARID1A expression in the pancreas is associated with reduced SOX9 expression and cancer cell differentiation [42]. Furthermore, Kimura et al. used a pancreatic cancer mouse model Ptf1a-Cre; Kras^G12D^ to show that the loss of ARID1A expression promotes tumorigenesis—from PanIN to ductal adenocarcinoma (PDAC)—and enhances intraductal papillary mucinous neoplasm (IPMN) formation from the duct cell. Following reference consultation, the authors selected SOX9 as the candidate duct cell differentiation factor. SOX9 expression was decreased in ARID1A-deficient IPMN, and this phenomenon was associated with decreased cell differentiation. However, SOX9 overexpression reversed this phenomenon. Moreover, decreased expression of ARID1A and SOX9 was observed in a subset of patients with IPMN. This result indicates that the mutational loss of ARID1A regulates pancreatic cell differentiation and cancer progression, providing clues for tumor development and progression in the pancreas.

ARID1A dysregulation plays an important role in GI cancers other than pancreatic cancer. ARID1A nonsense/missense/splice site mutations exist in patients with gastric cancer [14,15,16] and may serve to promote tumorigenesis [68]. The loss of ARID1A in vitro promotes cell proliferation [14,17] and E-cadherin-dependent migration and invasion [18]. ARID1A expression in the tumor of patients with pancreatic cancer decreased [17], and decreased ARID1A expression is associated with tumor stage/grade [17], lymphatic invasion/metastasis [19], and poor prognosis [17,19,20].

ARID1A nonsense/missense mutations are found in patients with liver cancer [23,24,25]. ARID1A increases the risk for cancer by promoting CYP450 (CYP2E1) transcriptional activation and reactive oxygen species (ROS) production in vivo, while decreasing metastasis via the transcriptional regulation of EMILIN1/MAT1A/LCN2/IL1R1 in vitro [26]. Conversely, ARID1A loss may increase the risk for steatohepatitis and cancer progression by altering immunity in vivo [27] or tumorigenesis by activating angiopoietin-2 (ANGPT2) transcription in vitro and causing angiogenesis in vivo [28]. These phenomena deserve further elucidation to clarify the context dependency of ARID1A mutation-regulated liver cancer progression, possibly using liver cancer mouse models [69,70].

ARID1A nonsense/missense/splice site mutations also exist in patients with cholangiocarcinoma [33,34,35,36] and are associated with a poor prognosis [37]. ARID1A (missense) mutations are found in patients with colon cancer [52,53,54], and ARID1A loss promotes invasive adenocarcinoma via SWI/SNF-dependent gene expression regulation in vivo [55] or proliferation/5-fluorouracil (5-FU) resistance in vitro [56]. Furthermore, ARID1A loss/decrement is associated with aging [57,58], poor tumor differentiation [56,58,59], tumor size [57], tumor grade [57,58], and metastasis [58] in human patients.

### 2.2. SMARCA4

SMARCA4 is an ATPase that provides energy to the SWI/SNF complex during chromatin remodeling [65] and is vital for development [71]. Similar to ARID1A, SMARCA4 is highly mutated in pancreatic cancer [45]. The in vivo loss of SMARCA4 results in the hypoplastic development of the pancreas [46] and enhances IPMN formation from the duct cell while suppressing PanIN formation from acinar cells carrying KRas^G12D^ mutation [46]. Furthermore, in terms of the duct cell, SMARCA4 loss in the early stage increases cancer dedifferentiation from IPMN to carcinoma; however, in the late stage, SMARCA4 overexpression enhances tumorigenicity by the promoter binding/transcriptional activation of high mobility group AT-hook 2 (HMGA2) and subsequent mesenchymal transition [47]. An in vivo inhibitor against bromodomain and extra-terminal (BET; a ~110 amino acid protein domain that recognizes acetylated lysine in gene induction; please see [72] for review on BET and its inhibitor JQ-1) suppresses SMARCA4 loss-induced HMGA2 expression and affects pancreatic tumorigenicity [47]. Moreover, in clinical specimens, SMARCA4 expression is decreased in IPMN but is increased in the carcinoma [46]. Therefore, targeting dysregulated epigenetic regulators with epigenetic therapeutics may offer a therapeutic benefit. Additionally, SMARCA4 missense mutations exist in human patients with gastric cancer [21] or cholangiocarcinoma [38], indicating its broad mutation spectra across multiple GI cancers.

### 2.3. SMARCA2

SMARCA2 is mutated in cancers of the stomach [21], liver [29], and pancreas [43] of human patients. SMARCA2 promotes pancreatic tumorigenesis via JAK2/STAT3 signaling in vitro and in vivo, with increased SMARCA2 correlating to advanced tumor stage and poor prognosis in human patients [44]. The diverse roles played by this ATPase family in various cancers deserve further elucidation to determine their clinical importance and therapeutic potential in different contexts.

## 3. Expression and Mutation of Histone Lysine Methylation Regulators in Pancreatic Cancer

Beside the positioning of the nucleosome by chromatin remodeling, covalent modification of DNA or histones plays an important role in the regulation of pancreatic cancer gene expression [61]. In addition to the modulators of histone acetylation [61] and arginine methylation [73], regulators of histone lysine methylation, such as demethylase KDM6A, methyltransferases KMT2C/KMT2D, and histone lysine methyltransferase, participate in pancreatic tumorigenesis and are frequently mutated. While the causes of mutations in these methylation regulators await elucidation, their importance and therapeutic potential [48,74,75] have been previously reported as below:

### 3.1. KDM6A

Lysine demethylase 6A (KDM6A) is a demethylase that targets H3K27me2 or H3K27me3 in complex proteins associated with Set1 (COMPASS), which is essential for development [76]. KDM6A also plays a vital role in embryogenesis [77], and its mutations are found in human patients with pancreatic cancer [48]. The in vivo loss of KDM6A enhances gender-specific squamous-like pancreatic cancer, as an X-chromosomal gene via super-enhancer activation can be targeted with a BET inhibitor [48] via KDM6A loss-regulated BRD4 function. Moreover, pancreatic cancer with KDM6A loss shows vulnerability to histone deacetylase (HDAC) inhibitor as KDM6A cooperates with histone acetyltransferase p300 (EP300) to regulate gene expression, and HDACi treatment reactivates p21 (CDKN1A) expression to restore cell cycle regulation in KDM6A-deficient pancreatic cancer in vitro [49]. Thus, BETi or HDACi may offer a synthetic lethality treatment strategy for pancreatic cancer upon KDM6A loss [48,49]; in this subset of cancer cells, a correlation between loss of KDM6A expression and poor prognosis was observed [49]. In addition, KDM6A nonsense/missense mutations exist in human patients with gastric cancer [22], suggesting its importance across various tumor types.

### 3.2. KMT2C and KMT2D

Lysine methyltransferase 2C and 2D (KMT2C and KMT2D) are monomethyltransferases in the COMPASS complex, both of which are target H3K4 for epigenetic regulation [22]. Moreover, the importance of KMT2D in development has been reported [50]. In addition to observing KMT2C (missense) mutations in patients with pancreatic cancer [50], gastric cancer [14], cholangiocarcinoma [39], or liver cancer [30], KMT2D (missense) mutations were found in patients with pancreatic cancer [50], liver cancer [31], or cholangiocarcinoma [38]. However, in vitro KMT2D depletion increases apoptosis and sensitivity toward 5-FU in pancreatic cancer [51]. This result is in accordance with RNA sequencing results that revealed downregulation in cell cycle and growth [78]. Furthermore, nonsense/missense mutations in KMT2C/D correlate with a better prognosis of patients with pancreatic cancer, as reported by Sausen et al. [50]. These results also suggest the necessity of performing a comprehensive study on the relationship between KMT2C/D mutation and its expression level and their impact on pancreatic cancer progression.

### 3.3. G9a

Other epigenetic modulators can also play roles in pancreatic cancer formation [78]. Regulators including euchromatic histone lysine methyltransferase 2 (EHMT2; G9a) that target H3K9 and H3K27, affect pancreatic cancer sensitivity toward gemcitabine via autocrine IL-8/CXCR1/2 stimulation in vitro [79], and further enhance mesenchymal transition by increasing polycomb repressive complex 2 (PRC2) recruitment, while decreasing the lysine demethylase 7A (KDM7A)’s expression to influence H3K27 methylation on E-cadherin promoter in vitro [80]. Furthermore, in colon cancer, G9a regulates cancer stem phenotype and chemoradioresistance via modulation of DNA damage response in vitro [81].

### 3.4. EZH2

Another histone lysine methyltransferase known as the enhancer of zeste homolog 2 (EZH2), also plays an important role in pancreatic cancer progression [82]. In vivo, EZH2 knockdown decreases pancreatic tumor growth and liver metastasis [83]. In clinical specimens, EZH2 expression was found to inversely correlate with E-cadherin expression and patient survival [82]. Moreover, a subset of patient-derived organoids displayed sensitivity toward EZH2 inhibition [84], suggesting the potential of EZH2 blockage for pancreatic cancer treatment, possibly in a model of personalized medicine based on an organoid platform. In addition, present synthetic lethality based on drug combinations, such as BET inhibitor plus poly (ADP-ribose) polymerase (PARP) inhibitor, has shown therapeutic efficiency in pancreatic cancer by inhibiting the BRD2/4-regulated DNA repair [85]. Hence, this study sheds light on the importance and necessity of investigating a therapeutic combination for treatment improvement with synthetic lethality strategy.

## 4. Therapeutics Targeting Epigenetic Regulators in Pancreatic Cancer

A synthetic lethal strategy can not only accentuate the efficacy of the cytotoxic effect, but may also decrease off-target side effects. Numerous studies have identified therapeutics causing alterations in the above epigenetic regulators in various cancer types (Table 3). For example, the loss of AIRID1A in ovarian cancer leads to increased expression of PI3K-interacting protein 1 gene (PIK3IP1), which is a negative regulator of PI3K-AKT signaling. EZH2 plays an antagonistic role in gene transcription, compared to AIRID1A. EZH2 inhibitor, GSK126, can upregulate the levels of PIK3IP1 upon the depletion of AIRID1A. Therefore, targeting EZH2 methyltransferase activity provides a personalized strategy for ARID1A-mutated cancers [86]. Moreover, HDAC2 downregulates PIK3IP1 expression. Blocking of HDAC2 induces apoptosis in ARID1A-inactivated cells. Other studies have indicated that HDAC6 inhibition could trigger cell apoptosis through p53 activation. In this way, HDAC inhibitors could be another anti-cancer agent for treating ARID1A-mutated cancers [87,88]. Furthermore, the tyrosine kinase inhibitor dasatinib mediates apoptosis by targeting YES1 inhibition in ARID1A-null tumor cells [89]. Mismatch repair (MMR) deficiency causes a molecular feature of microsatellite instability (MSI) and may contribute to response toward immune-checkpoint blockade. A prior study demonstrated that ARID1A interacts with MMR-associated protein MSH2 at the damage site. Therefore, ARID1A deficiency also contributes to providing a potential synthetic lethal strategy for targeting programmed death-ligand 1 (PD-L1; CD274) [90]. Through a high-throughput screening, targeting the BET protein with its specific inhibitor JQ1 inhibits the growth of ARID1A-mutant cells [91]. In colon cancer, ARID1A-deficient cells exhibit reduced expression of topoisomerase 2A and a decatenation defect, which render tumor cells sensitive to an ATR serine/threonine kinase inhibitor [92] or PARP inhibitor [93].

SMARCA2/4 deficiency leads to EZH2 dependency for cell survival. Therefore, EZH2i could be a therapeutic target in SMARCA2/4-impaired cells [94]. SMARCA4 loss results in low levels of cyclin-dependent kinase 4/6 (CDK4/6) inhibitor p16INK4a in lung cancer. This phenomenon suggests that SMARCA4-deficient cell lines are sensitized to CDK4/6 inhibitors [95]. Through RNAi-mediated depletion or chemical inhibition screening, aurora kinase A (AURKA) inhibition induces apoptosis and cell death in SMARCA4-deficient cells in vitro and in xenograft mouse models [96]. KDM6A mutation can be targeted with EZH2i in bladder cancer and multiple myeloma (MM), due to EZH2 dependency for survival [97,98].

**Figure 2:**
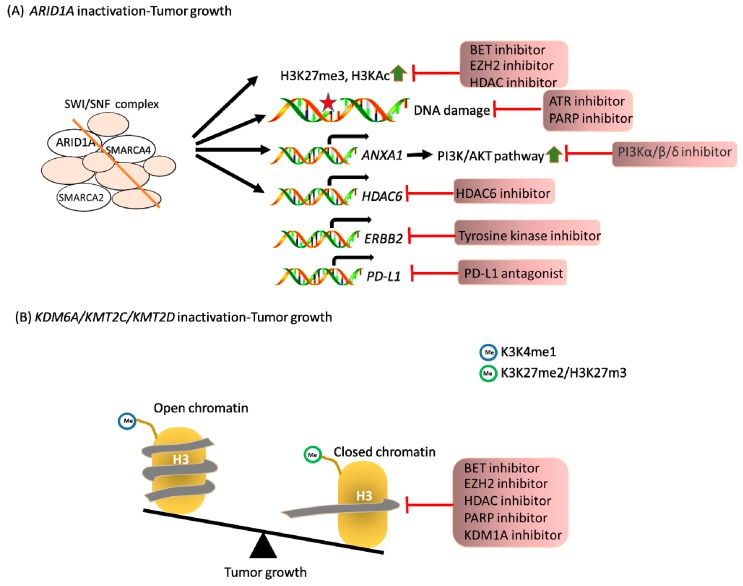
Loss of ARID1A, KDM6A, KMT2C, and KMT2D triggers synthetic lethal opportunities for cancer treatment. (**A**) ARID1A is a member of the SWI/SNF chromatin-remodeling complex that regulates transcription and nucleosome condensation. The blockage of specific oncogenic pathways was defined as a potential strategy in ARID1A-deficient cells. (**B**) KDM6A and KMT2C/KMT2D represent lysine demethylase and methyltransferase, respectively. The inactivation of KDM6A, KMT2C, and KMT2D is associated with a closed chromatin structure that is specifically sensitized by the indicated inhibitors.

KDM6A is most frequently mutated in myelodysplastic syndrome (MDS) and acute myeloid leukemia (AML). Using an epigenetic drug library screening in KDM6A-null cells, an inhibitor against lysine demethylase 1A (KDM1A; LSD1) could be a novel strategy to specifically inhibit the growth of KDM6A-deficient cells [99]. In epithelial cancers, KMT2C deficiency displays defects of homologous recombination-mediated DNA double-strand break repair that triggers PARPi sensitization of cells [100]. Moreover, in pancreatic cancer, KDM6A loss can be targeted using BETi through 78 small-molecule inhibitors [48]. Moreover, as reviewed by Xu et al. [72], the BET protein family—including BRD2, BRD3, BRD4, and BRDT—recognizes acetylated lysine on histone and transcription factors and promotes transcription with their bromodomain or extraterminal domain. The role of the BET family in tumorigenesis was implied by the promotion of liver cancer progression by BRD4 [32]. Due to the importance of bromodomain in transcriptional regulation, BET inhibitors, including diazepines JQ1 and iBET that target this domain, have been developed. Even though bromodomains from different sites on the same BET protein or from different BET proteins display diverse functions, these inhibitors still exhibit anti-tumor activity in vivo [48,72,85,86], indicating their therapeutic potential for cancer treatment. Although contextual differences exist across cancer types, the above-mentioned therapeutics identified with bioinformatics or screening strategies [48] are promising alternatives to treat tumors with synthetic lethal strategies in *ARID1A*, *SMARCA4*, *KDM6A*, *KMT2C*, or *KMT2D* with GI cancers as a field of future research interest (Figure 2).

## 5. Conclusions

In the present review, we summarized the mutations of epigenetic regulators, including *ARID1A*, *SMARCA2*, *SMARCA4*, *KDM6A*, *KMT2C*, *KMT2D*, and *BRD4* in GI cancers—in particular, pancreatic cancer—and found these regulators to be frequently mutated. The screening of tumor cells by utilizing chemicals, siRNAs, and shRNAs helps to identify therapeutic targets in epigenetic regulator-mutated cancers. However, the context dependency for each neoplasia needs further examination for the possible broad-spectrum application of the therapeutics mentioned in Table 3 across multiple tumor types. Accordingly, the screening strategy that targets multiple cancer types with a mutation in the same epigenetic regulator (for example, the ARID1A mutation in ovarian and colon tumors) may aid in the identification of therapeutics. This strategy could help advance the treatment of epigenetic regulator-mutated cancers.

## Figures and Tables

**Table 1:** Aliases of *ARID1A*, *SMARCA2*, *SMARCA4*, *KDM6A*, *KMT2C, KMT2D*, *BRD2*, *BRD3*, *BRD4*, *BRDT*.

Gene Symbol	Gene Name	Alias
*ARID1A*	AT-rich interaction domain 1A	*B120, BAF250, BAF250a, BM029, C1orf4, CSS2, ELD, MRD14, OSA1, P270, SMARCF1, hELD, hOSA1*
*SMARCA2*	SWI/SNF related, matrix associated, actin dependent regulator of chromatin, subfamily a, member 2	*BAF190, BRM, NCBRS, SNF2, SNF2L2, SNF2LA, SWI2, Sth1p, hBRM, hSNF2a*
*SMARCA4*	SWI/SNF related, matrix associated, actin dependent regulator of chromatin, subfamily a, member 4	*BAF190, BAF190A, BRG1, CSS4, MRD16, RTPS2, SNF2, SNF2L4, SNF2LB, SWI2, hSNF2b*
*KDM6A*	lysine demethylase 6A	*KABUK2, UTX, bA386N14.2*
*KMT2C*	lysine methyltransferase 2C	*HALR, KLEFS2, MLL3*
*KMT2D*	lysine methyltransferase 2D	*AAD10, ALR, CAGL114, KABUK1, KMS, MLL2, MLL4, TNRC21*
*BRD2*	bromodomain containing 2	*BRD2-IT1, D6S113E, FSH, FSRG1, NAT, O27.1.1, RING3, RNF3*
*BRD3*	bromodomain containing 3	*ORFX, RING3L*
*BRD4*	bromodomain containing 4	*CAP, HUNK1, HUNKI, MCAP*
*BRDT*	bromodomain testis associated	*BRD6, CT9, SPGF21*

**Table 2:** *ARID1A*, *SMARCA2*, *SMARCA4*, *KDM6A*, *KMT2C*, *KMT2D*, and *BRD4* mutation status in cancers of the stomach, liver, biliary duct, pancreas, and colon.

Cancer	Gene	Mutation	Expression	Effect	Reference
Stomach	*ARID1A*	Nonsense, missense, splice site			[14,15,16]
			Loss	Increased proliferation	[14,17]
			Loss	Increased migration and invasion	[18]
			Loss	Association with tumor stage and grade	[17]
			Loss	Association with lymphatic invasion and lymph node metastasis	[19]
			Loss	Association with poor prognosis	[17,19,20]
	*SMARCA2*	Mutation			[21]
	*SMARCA4*	Missense			[21]
	*KDM6A*	Nonsense, missense			[22]
	*KMT2C*	Mutation			[14]
Liver	*ARID1A*	Nonsense, missense			[23,24,25]
			Loss	Decreased tumorigenesis; increased metastasis	[26]
			Loss	Increased steatohepatitis and tumorigenesis	[27]
			Loss	Increased tumorigenesis and angiogenesis	[28]
	*SMARCA2*	Missense			[29]
	*KMT2C*	Mutation			[30]
	*KMT2D*	Mutation			[31]
	*BRD4*		Gain	Increased tumorigenesis	[32]
Biliary duct	*ARID1A*	Nonsense, missense, splice site			[33,34,35,36]
		Nonsense, missense		Association with poor prognosis	[37]
	*SMARCA4*	Missense			[38]
	*KMT2C*	Mutation			[39]
	*KMT2D*	Mutation			[38]
Pancreas	*ARID1A*	Nonsense, missense			[40,41]
			Loss	Decreased differentiation	[42]
	*SMARCA2*	Mutation			[43]
			Gain	Decreased patient survival and drug sensitivity	[44]
	*SMARCA4*	Mutation			[45]
			Loss	Increased IPMN; decreased PanIN	[46]
			Loss	Decreased late stage tumorigenicity	[47]
	*KDM6A*	Mutation			[48]
			Loss	Increased squamous-like cancer	[48]
			Loss	Decreased overall/recurrence-free survival	[49]
	*KMT2C*	Missense			[50]
	*KMT2D*	Missense			[50]
			Loss	Increased apoptosis and drug sensitivity	[51]
Colon	*ARID1A*	Missense			[52,53,54]
			Loss	Increased aggressive adenocarcinoma	[55]
			Loss	Increased proliferation and drug resistance	[56]
			Loss	Association with ageing	[57,58]
			Loss	Association with poor tumor differentiation	[56,58,59]
			Loss	Association with tumor size	[57]
			Loss	Association with tumor grade	[57,58]
			Loss	Association with metastasis	[58]

**Table 3:** Therapeutic targets in cancers with alterations in *ARID1A, SMARCA2, SMARCA4, KDM6A, KMT2C,* or *KMT2D*.

Gene	Cancer	Therapeutic Target	Reference
*ARID1A*	Ovary	EZH2	[86]
		Pan HDAC	[87]
		HDAC6	[88]
		tyrosine kinases	[89]
		CD274	[90]
		BET	[91]
	Colon	ATR	[92]
		PARP	[93]
*SMARCA2*	Ovary	EZH2	[94]
*SMARCA4*	Lung	CDK4/6	[95]
		AURKA	[96]
	Ovary	EZH2	[94]
*KDM6A*	Bladder	EZH2	[97]
	Multiple myeloma	EZH2	[98]
	Acute myeloid leukemia	KDM1A	[99]
	Pancreas	BET	[48]
		HDAC	[49]
*KMT2C*	Epithelial cancer	PARP	[100]
